# The impact of fasciotomy on inpatient outcomes in lower leg fracture management

**DOI:** 10.1007/s00590-023-03666-z

**Published:** 2023-08-03

**Authors:** Anne Sophie Mittlmeier, Hans-Christoph Pape, Valentin Neuhaus, Claudio Canal

**Affiliations:** Division of Trauma Surgery, University Hospital Zurich (USZ), University of Zurich (UZH), Raemistrasse 100, 8091 Zurich, Switzerland

**Keywords:** Fasciotomy, Lower extremity fracture, Clinical outcome

## Abstract

**Background:**

While lower extremity fractures are common injuries, concomitant compartment syndrome can lead to significant implications and surgical release (fasciotomy) is essential. The aim of this study was to identify potential predictors of compartment release and risk factors related to complications. Using a large nationwide cohort, this study compared patients suffering from lower extremity fractures with and without compartment syndrome during their primary in-hospital stay following trauma.

**Methods:**

A retrospective analysis was conducted using the prospective surgical registry of the working group for quality assurance in surgery in Switzerland, which collects data from nearly 85% of all institutions involved in trauma surgery. *Inclusion criteria* Patients who underwent surgical treatment for tibia and/or fibula fractures between January 2012 and December 2022 were included in the study. *Statistics* Statistical analysis was performed using Chi-square, Fisher’s exact test, and *t* test. Furthermore, a regression analysis was conducted to determine the independent risk factors for fasciotomy and related complications. In the present study, a *p* value less than 0.001 was determined to indicate statistical significance due to the large sample size.

**Results:**

The total number of cases analyzed was 1784, of which 98 underwent fasciotomies and 1686 did not undergo the procedure. Patients with fasciotomies were identified as significantly younger (39 vs. 43 years old) and mostly male (85% vs. 64%), with a significantly higher American Society of Anesthesiologists (ASA) score (ASA III 10% vs. 6%) and significantly more comorbidities (30% vs. 20%). These patients had significantly longer duration of surgeries (136 vs. 102 min). Furthermore, the total number of surgical interventions, the rate of antibiotic treatment, and related complications were significantly higher in the fasciotomy group. Sex, age, comorbidities, and fracture type (both bones fractured) were identified as relevant predictors for fasciotomy, while ASA class was the only predictor for in-hospital complications. *Outcomes* Patients who underwent fasciotomy had a significantly longer hospital stay (18 vs. 9 days) and a higher complication rate (42% vs. 6%) compared to those without fasciotomy. While fasciotomy may have played a role, other factors such as variations in patient characteristics and injury mechanisms may also contribute. Additionally, in-house mortality was found to be 0.17%, with no patient death recorded for the fasciotomy group.

**Conclusions:**

Fasciotomy is vital. The knowledge about the further course is, however, helpful in resource allocation. We found significant differences between patients with and without fasciotomy in terms of age, sex, complication rate, length of stay, comorbidities, duration of operations, and use of antibiotics during their primary in-hospital stay. While the severity of the underlying trauma could not be modulated, awareness of the most relevant predictors for fasciotomy and related complications might help mitigate severe consequences and avoid adverse outcomes.

## Introduction

Lower extremity fractures—one of the most common fractures observed in trauma patients [1]—are closely associated with increased morbidity due to severe soft tissue involvement and mortality, especially in elderly people [2]. The reasons for such fractures are manifold, ranging from sports injuries, falls from a height, and road traffic accidents to low-energy trauma [3].

Compartment syndrome of the lower limb manifests most frequently in the lower leg and is often, but not exclusively, associated with high-energy injuries [4]. It may also develop secondarily following surgical fracture treatment involving excessive postoperative swelling.

Depending on the interval between the onset of compartment syndrome and successful compartment release through fasciotomy, poor functional outcome may be the consequence of increased rates of complications, e.g., surgical site infection, nonunion, loss of vital muscles following muscle fibrosis, or even necrosis [5]. Independent from this, postoperative complications can not all be ascribed to the fasciotomy itself, it is rather a multifactorial pathogenesis. Evidently, an early diagnosis of compartment syndrome is crucial for the prevention of adverse events. In this context, the literature shows that the age of the patient is a significant predictive parameter for the outcome of lower extremity fractures with concomitant compartment syndrome. As such, incipient or manifest compartment syndrome can be assessed as the harbinger of a problematic clinical course in treating lower leg fractures [6].

To the best of our knowledge, available data on the in-hospital outcomes of patients undergoing fasciotomy for lower extremity fracture treatment are scarce. Addressing this gap, the aim of this study was to evaluate the extent to which compartment syndrome is a significant predictor of in-hospital complications and length of stay for lower extremity fractures, as recorded by a nationwide quality management tool.

## Materials and methods

The current study retrospectively analyzed the data of lower leg fracture patients with and without fasciotomy obtained from the prospective surgical registry of the working group for quality assurance in surgery (“Arbeitsgemeinschaft für Qualitätssicherung in der Chirurgie” or AQC). The AQC is a voluntary association of physicians in Switzerland formed with the aim of evaluating the in-hospital outcomes of surgical patients. More than 80 Swiss surgical departments, representing 85% of all such departments in Switzerland, contribute the clinical data of hospitalized patients to the AQC. Having been active for more than 25 years, the tool comprises about 2 million anonymized cases with the purpose of ensuring surgical quality in Switzerland [7]. Notably, according to the authorization requirements of the local cantonal ethical review board (Zurich, Switzerland), no institutional review board approval was required to utilize this data.

### Inclusion criteria

The inclusion criteria for the current study were patients with operatively treated tibia and/or fibula fractures. The International Classification of Diseases, Tenth Edition (ICD-10)—a clinical cataloging system developed by the World Health Organization (WHO) [8]—was employed to identify the cohort. The ICD-10 codes that were considered in this study were version 2019 S82.2—encoding fracture of a shaft of the tibia, S82.21—encoding fracture of tibial shaft along with fracture of fibula (any part), and S82.28—encoding other fracture variants of the tibial shaft. Meanwhile, the study excluded 523 cases that were either characterized by incomplete data or those that did not necessitate surgical intervention, as well as cases that did not involve initial care, for example, those concerning hardware removal.

### Exclusion criteria

Patients with missing or incomplete information in their medical records were excluded from the study. Cases that did not require surgical intervention for the tibia and/or fibula fractures were excluded since this study focused specifically on the outcomes of patients who underwent surgical treatment for their fractures. Patients who presented for reasons other than initial care, such as hardware removal or follow-up appointments, were excluded from the study. The focus of this study was on the in-hospital outcomes of patients receiving their initial surgical treatment. Patients with fractures resulting from underlying pathological conditions, such as bone tumors or metabolic bone diseases, were also excluded. For the determination of the predictors, we excluded the ASA IV group due to its small sample size (*n* = 10).

In this study, complications were categorized into three distinct groups. The first group comprised intraoperative complications, which encompassed surgery-specific events such as injuries to veins and nerves. The second group included postoperative complications, which occurred after the surgical intervention and consisted of issues such as rotation errors, implant malposition, wound healing difficulties, and hematomas. Lastly, the third group consisted of general complications that arose during the patients’ hospitalization, encompassing conditions such as pneumonia, allergic reactions, urinary tract infections, and urinary retention.

We defined as outcome criteria the duration of the procedure and the hospitalization as well as the percentage of patients who experienced complications for patients who underwent fasciotomy compared to those who did not. The complications were divided in intraoperative, postoperative and general complications.

Finally, a total of 1784 patients with lower leg fractures treated between January 1, 2012, and December 31, 2022, were analyzed, among whom 98 had undergone fasciotomy and 1686 had not. The following parameters from the AQC database were considered for study: patient’s age at admission, sex, and American Society of Anesthesiologists (ASA) score. The ASA physical status classification system assesses the patient´s pre-anesthesia medical comorbidities [9].

Moreover, to compare the groups with and without fasciotomies, the following information were taken into consideration: type of admission (emergency or planned), type of insurance (statutory or private), length of hospital stay in days, comorbidities as chronic lung disease, neurologic or psychiatric disorders, diabetes mellitus, hypertensive disease, cardiac insufficiency, previous myocardial infarction and malignant diseases, discharge status, surgeon class (senior attending, junior attending, resident), duration of surgery in minutes, teaching of the procedure, thromboembolism, antibiotics prescribed during hospital stay, complications, number of operations, and the initially performed operation (external fixator, closed reduction and internal fixation, open reduction and internal fixation).

### Statistics

The AdjumedAnalyze (Adjumed Services AG, Zurich, Switzerland) software was utilized to download the data from the AQC database. Furthermore, SPSS version 29 (IBM Corp., Armonk, NY, USA) was used to statistically analyze the data. Furthermore, chi-square and Fisher’s exact tests were employed for analysis of the categorical data, which were presented as numbers of patients and their corresponding percentages. Furthermore, an unpaired *t *test was implemented to compare the numerical data of the two groups. The results were presented as means ± standard deviation. Due to the large number of patients, a *p* value of < 0.001 was considered statistically significant. Furthermore, a *p* value of 0.001–0.049 indicated suggestive evidence. Finally, the study utilized binary logistic regression as a statistical tool to explore and identify independent risk factors associated with the likelihood of complications and the need for fasciotomy.

## Results

### Population

Among the 1784 patients included in this study, those who had undergone fasciotomy were significantly younger than those without fasciotomy (39 vs. 43 years), mostly men (85% vs. 64%), and had a significantly higher ASA score (ASA III 10% vs. 6%). As expected, patients who underwent fasciotomy were invariably admitted to emergency departments (100% vs. 96%). Moreover, fasciotomy was found to be more frequently associated with fractures of both tibia and fibula fractures than in isolated fibula or tibia fractures. In over 81% of the patients, fasciotomy was performed within 24 h after admission. However, in 18 cases, compartment syndrome was diagnosed and treated 24 h after admission.

The type of insurance was similar for both groups, with 77% of the patients having statutory insurance. Additionally, patients with fasciotomies had significantly more comorbidities (30% vs. 20%) (Table 1).

**Table 1:** Type of admission; patient characteristics

Parameter		Total (*n* = 1784)		Group without fasciotomy (*n* = 1686)		Group with fasciotomy (*n* = 98)		*p* value
		*n*	%	*n*	%	*n*	%	
Age (years)	Mean ± SD	43 ± 20		43 ± 20		39 ± 19		0.036
Sex	Male	1159	65.0	1076	63.8	83	84.7	< 0.001
	Female	625	35.0	610	36.2	15	15.3	
ASA	I (Healthy person)	989	55.4	940	55.8	49	50.0	0.002
	II (Mild systemic disease)	672	37.7	636	37.7	36	36.7	
	III (Severe systemic disease)	113	6.3	103	6.1	10	10.2	
	IV (Severe systemic disease that is a constant threat to life)	10	0.56	7	0.42	3	3.1	
Type of admission	Emergency	1718	96.3	1620	96.1	98	100	0.046
	Registered, planned, other and unknown	66	3.7	66	3.9	0	0	
Insurance	Statutory	1366	76.6	1292	76.6	74	75.5	0.799
	Private	418	23.4	394	23.4	24	24.5	
Length of stay (days)	Mean ± IQR	9 ± 6		8.5 ± 5		18 ± 10		< 0.001
Comorbidity	Yes	364	20.4	335	19.9	29	29.6	0.020
Discharge	Deceased	3	0.17	3	0.18	0	0	0.137
	At Home	1476	82.7	1399	83.0	77	78.6	
	Rehabilitation clinic	123	6.9	109	6.5	14	14.3	
	Nursing home	29	1.6	28	1.7	1	1.0	
	Old people’s home	30	1.7	29	1.7	1	1.0	
	Other/unknown	84	4.7	81	4.8	3	3.1	
	Other hospital	39	2.2	37	2.2	2	2.0	
Diagnosis	S82.2 Fracture of tibial shaft	92	5.2	92	5.5	0	0	0.002
	S82.21 Fracture of tibial shaft: with fracture of fibula (any part)	1142	64.0	1064	63.1	78	79.6	
	S82.28 Fracture of tibial shaft: other	550	30.8	530	31.4	20	20.4	

*SD* standard deviation; *ASA* American Society of Anesthesiologists classification system; *IQR* interquartile range

### Procedures

**Fig. 1:**
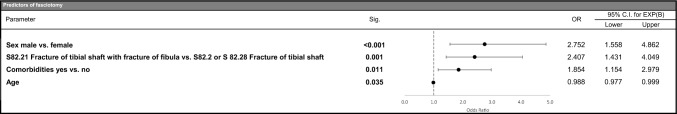
Predictors of fasciotomy. *Sig *Significance; *OR* Odds Ratio

In the case of patients who underwent fasciotomy, the duration of the main surgical intervention was significantly longer (136 vs. 102 min), and they also had a higher number of operations (3.7 vs. 1.2) during hospitalization. Furthermore, in both groups, most of the stabilization techniques were performed through closed reduction and internal fixation (47%), followed by open reduction and internal fixation (40%), while an external fixator was considered in rare cases (12%) (Table 2). The significant predictors for fasciotomy were male sex (OR = 2.752), diagnosis of a combined fracture of the fibula and the tibia, presence of comorbidities, and young age (Fig. 1).

**Table 2:** Type of anesthesia; procedure characteristics

Parameter		Total (*n* = 1784)		Group without fasciotomy (*n* = 1686)		Group with fasciotomy (*n* = 98)		*p *value
		*n*	%	*n*	%	*n*	%	
Surgeon class	Senior attending	706	39.6	670	39.7	36	36.7	0.837
	Junior attending	735	41.2	693	41.1	42	42.9	
	Resident	343	19.2	323	19.2	20	20.4	
Duration surgery (minutes)	Mean ± IQR	104 ± 66		102 ± 64		136 ± 89		< 0.001
Teaching	Yes	676	37.9	634	37.6	42	42.9	0.297
Thromboembolism prophylaxis during hospital stay	No thromboembolism prophylaxis	338	18.9	311	18.4	27	27.6	0.041
	Thromboembolism prophylaxis	1220	68.4	1156	68.6	64	65.3	
	Anticoagulation	23	1.3	21	1.2	2	2.0	
	Unknown	203	11.4	198	11.7	5	5.1	
Antibiotics during hospital stay	No antibiotics	72	4.0	68	4.0	4	4.1	< 0.001
	Prophylactic antibiotics	1537	86.2	1466	87.0	71	72.4	
	Antibiotic therapy	175	9.8	152	9.0	23	23.5	
Complication	Yes	142	8.0	101	6.0	41	41.8	< 0.001
Operation	External fixator	221	12.4	198	11.7	23	23.5	0.001
	Closed reduction, internal fixation	846	47.4	800	47.4	46	46.9	
	Open reduction, internal fixation	717	40.2	688	40.8	29	29.6	
Number of operations	Mean ± SD	1.3 ± 0.98		1.2 ± 0.55		3.7 ± 2.6		< 0.001

*IQR* interquartile range; *SD* standard deviation

### Outcomes

In addition to the characteristics identified above, patients who underwent fasciotomy stayed at the hospital for a significantly longer period than those without fasciotomy (18 vs. 9 days). Notably, while complications were reported in 8% (*n* = 142) of the patients from both groups, the group with fasciotomy exhibited a significantly higher complication rate than the group without it (42% vs. 6%).

Meanwhile, the most commonly reported intraoperative complications were injuries to the veins and nerves. Postoperative complications included rotation error, implant malposition, wound healing issues, and hematoma. Compartment syndrome was counted as a complication in 16 cases, alongside other additional complications. Apart from this, the general complications reported were pneumonia, allergic reaction, urinary tract infection, and urinary retention.

**Fig. 2:**
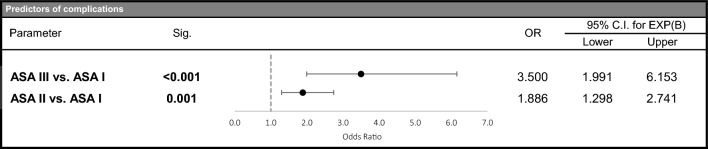
Predictors of complications. *Sig *Significance; *OR* Odds Ratio

The predictor for complications was a high ASA score (ASA III vs. I, with OR = 3.500, and ASA II vs. I, with OR = 1.886) (Fig. 2). In the case of both groups, most of the patients were discharged following treatment (83%). Additionally, in-house mortality was found to be 0.17%, with no patient death recorded for the fasciotomy group.

### Teaching and surgeon class

Most of the operations were performed by junior attending surgeons (41%), followed by senior attending surgeons (40%), and then residents (19%), with no significant difference between the two groups. Compared to operations that did not involve fasciotomies (41%), surgical treatments involving fasciotomies were performed more often by junior attendants (43%). Moreover, teaching was not found to be associated with a negative outcome.

## Discussion

The goal of the present study was to investigate the extent to which compartment syndrome of the lower leg in conjunction with a lower leg fracture impacts the essential parameters of primary in-hospital stay and the clinical course of treatment. One of the main findings of our cohort study was that around 6% of patients with lower leg fractures undergo fasciotomy. Furthermore, the patient being male, of a younger age, having a notable presence of comorbidities, and a complete fracture of the lower leg were identified as relevant predictors of the need to undergo fasciotomy. Moreover, patients with fasciotomy were found to have experienced three times more operations, two times longer duration of hospital stay, and seven times as many complications than those who did not undergo the procedure. With regard to in-house complications, a higher ASA score was identified as the most significant predictor.

In high-energy and complex trauma, the need for fasciotomy in the lower leg could increase to 50% [10]. A frequency of 5.8%, as observed in our study, points to the fact that both low- and high-energy trauma cases were considered, which reflects the distinct character of this cohort study based on a nationwide database comprising 85% of all Swiss hospitals performing acute trauma surgery.

Consistent with Park et al., this study confirmed that patients with fasciotomies are mostly young men, which underlines the fact that young males are more frequently involved in high-energy trauma than elderly people [11]. Correspondingly, the high number of patients with complete lower leg fractures needing fasciotomy indicates that the amount of energy involved in the trauma strongly correlates with the need for fasciotomy. Interestingly, higher degrees of comorbidities, as reflected by ASA classes III and IV—usually observed in the elderly population—were associated with a higher rate of compartment release. This may be linked to the findings in the literature that claim elderly patients may develop compartment syndrome as a result of their intake of oral anticoagulants, such as Apixaban [12].

Not surprisingly, the duration of surgery in the case of patients with fasciotomy was significantly longer compared to cases without fasciotomy, which aligns with the finding that prolonged operating time is probably a marker of technical difficulty and lengthened exposure of the wound. Such circumstances, in conjunction with more severe soft tissue trauma, are more likely to lead to higher rates of postoperative infections [13]. This might explain the significantly higher rates of antibiotic use by patients undergoing fasciotomy during their hospital stay. Furthermore, this could be linked to longer in-hospital stays observed for patients after fasciotomy, as confirmed by other researchers [14]. In the present study, primary in-hospital stay by patients following fasciotomy was twice as long compared to those who did not undergo the procedure. Moreover, a surgical intervention rate of almost four surgeries in the case of fasciotomy indicates that a larger number of such patients required more than a secondary operation for soft tissue closure, e.g., serial debridement, step-wise approximation of the fasciotomy wound, or conversion from external to internal fixation. Data about the conversion of fixations may be derived from the primary rate of the use of external fixators, which was twice as high for the group with fasciotomy (23%) compared to the group without it (12%).

This is, in particular, relevant especially from the logistical view of clinics, which are confronted with concurrent problems like bed shortage to be able to expect a higher effort with patients of the fasciotomy group.

There are many factors that increase the likelihood for fasciotomy [15]. Combined plateau-shaft fractures, presence of fibula fracture, Schatzker VI fractures, OTA/AO 41-C2, and 41-C3 fractures were predictive in the literature of acute compartment syndrome [16, 17]. However, our cohort lacked detailed fracture classifications.

Saiz et al. showed that multiple comorbidities, including smoking, drug use, and alcohol use, were associated with an increased risk of having a fasciotomy. This may be due to these patients having decreased sensorium, which can make the diagnosis of an acute compartment syndrome diagnosis difficult; it may also be from vasoconstriction of smaller vessels; this leads to decreased perfusion and earlier myonecrosis [18]. A similar effect may be caused by state of shock on admission as a cause of more swelling. Our dataset was limited and did not offer in-depth insights into patients’ medical history, comorbidities, or the specific circumstances surrounding their conditions upon admission to the hospital.

Furthermore, complication rates were found to be significantly higher in the case of fasciotomy—found in almost every second patient who underwent the procedure—compared to cases without fasciotomy. Consequently, in-depth knowledge of the predictors of complications arising from fasciotomy may be crucial for prevention and early control. The current study observed a high ASA class to be the most crucial predictor, apart from young and, in general, healthy male patients with relevant comorbidities, of a high-risk cohort in terms of developing complications. The literature also emphasizes that significantly higher ASA scores and, in turn, detection of more comorbidities represents an increased risk factor for having a fasciotomy [19].

However, while employing the AQC database to collect and interpret data has its advantages, this methodology also suffers from a few inherent weaknesses. One of the most significant advantages of utilizing this extant database is the availability of a large amount of data from all levels of hospitals across Switzerland, accounting for a majority of the patients treated from January 2012 to December 2022. However, a major limitation of this dataset is that it registers only primary in-hospital data. As a result, no data on long-term effects, functional and final outcomes, or late complications that arose after the patients’ discharge from the hospital could be accounted for in this study. In addition to that the AQC database did not allow to further differentiate the specific in-hospital complications like surgery specific associated events or injuries to veins and nerves.

Furthermore, a potential bias in this study could be distortion of data by physicians based on their interpretation of the questions in the AQC datasheet. For example, the current study found a significantly high complication rate in patients treated with fasciotomies. Although this is consistent with the findings reported in the literature [20], the compartment syndrome itself might already be registered as a complication.

## Conclusion

Consistent with previous findings reported in the literature, this study analyzed a large volume of data encompassing 11 years from a nationwide quality assessment tool to confirm that patients who underwent fasciotomies after a fracture to their lower leg were mostly young men with comparatively more comorbidities who underwent longer operations and frequently developed complications, as well as had a longer primary stay at the hospital. Most importantly, a higher ASA class was identified as the only predictor of complications.

However, since the findings of this study are drawn from primary in-hospital observations, further research should analyze long-term complications and functional outcomes as the next step in evaluating the long-term validity of the predictors identified in this study.

## References

[1] Wu AM, Bisignano C, James SL, Abady GG, Abedi A, Abu-Gharbieh E, Alhassan RK, Alipour V, Arabloo J, Asaad M, Asmare WN (2021). Global, regional, and national burden of bone fractures in 204 countries and territories, 1990–2019: a systematic analysis from the global burden of disease study 2019. Lancet Healthy Longev.

[2] Somersalo A, Paloneva J, Kautiainen H, LÖNnroos E, HEinÄNen M, Kiviranta I (2016). Increased mortality after lower extremity fractures in patients < 65 years of age. Acta Orthop.

[3] Lee C, Porter KM (2005). Prehospital management of lower limb fractures. Emerg Med J.

[4] Frink M, Hildebrand F, Krettek C, Brand J, Hankemeier S (2010). Compartment syndrome of the lower leg and foot. Clin Orthop Relat Res.

[5] Köstler W, Strohm PC, Südkamp NP (2005). Acute compartment syndrome of the limb. Injury.

[6] Schmidt AH (2011). The impact of compartment syndrome on hospital length of stay and charges among adult patients admitted with a fracture of the tibia. J Orthop Trauma.

[7] Disziplinen AfQidc (2023) AQC-Gemeinschaft FAQ: AQC. https://aqc.ch/aqc-gemeinschaft/faq/

[8] WHOrganization (2023) WHO International Statistical Classification of Diseases and Related Health Problems (ICD). https://www.who.int/standards/classifications/classification-of-diseases

[9] Anestesiologists ASo. ASA Physical Status Classification System 2020. https://www.asahq.org/standards-and-guidelines/asa-physical-status-classification-system

[10] Bowyer MW (2015). Lower extremity fasciotomy: indications and technique. Curr Trauma Rep.

[11] Park S, Ahn J, Gee AO, Kuntz AF, Esterhai JL (2009). Compartment syndrome in tibial fractures. J Orthop Trauma.

[12] Adams J, Ramos JH, Appareddy S, Nguyen HA, Garcia G (2022). Acute lower-extremity posterior compartment syndrome: a rare complication of apixaban use. Ann Inter Med Clin Cases.

[13] Colman M, Wright A, Gruen G, Siska P, Pape HC, Tarkin I (2013). Prolonged operative time increases infection rate in tibial plateau fractures. Injury.

[14] Field CK, Senkowsky J, Hollier LH, Kvamme P, Saroyan RM, Rice JC (1994). Fasciotomy in vascular trauma: is it too much, too often?. Am Surg.

[15] Tillinghast CM, Gary JL, Mauffrey C, Hak DJ, Martin IM (2019). Compartment syndrome of the lower extremity. Compartment syndrome: a guide to diagnosis and management.

[16] Marchand LS, Working ZM, Rane AA, Elliott IS, Gilbertson E, Rothberg DL (2020). Compartment syndrome in tibial plateau fractures: do previously established predictors have external validity?. J Orthop Trauma.

[17] Wang T, Guo J, Long Y, Hou Z (2023). Predictors of acute compartment syndrome in patients with tibial fractures: a meta-analysis. Int Orthop.

[18] Ouellette EA (1998). Compartment syndromes in obtunded patients. Hand Clin.

[19] Saiz AM, Wellman AC, Stwalley D, Wolinsky P, Miller AN (2020). The incidence and risk factors associated with the need for fasciotomy in tibia and forearm fractures: an analysis of the national trauma data bank. J Orthop Trauma.

[20] Velmahos GC, Theodorou D, Demetriades D, Chan L, Berne TV, Asensio J (1997). Complications and nonclosure rates of fasciotomy for trauma and related risk factors. World J Surg.

